# The Minimal and Short-Lived Effects of Minority Language Exposure on the Executive Functions of Frisian-Dutch Bilingual Children

**DOI:** 10.3389/fpsyg.2017.01453

**Published:** 2017-08-29

**Authors:** Evelyn Bosma, Eric Hoekstra, Arjen Versloot, Elma Blom

**Affiliations:** ^1^Fryske Akademy, Royal Netherlands Academy of Arts and Sciences Leeuwarden, Netherlands; ^2^Amsterdam Center for Language and Communication, University of Amsterdam Amsterdam, Netherlands; ^3^Leiden University Centre for Linguistics, Leiden University Leiden, Netherlands; ^4^Department of Modern Foreign Languages and Cultures, University of Amsterdam Amsterdam, Netherlands; ^5^Special Education: Cognitive and Motor Disabilities, Department of Education and Pedagogy, Utrecht University Utrecht, Netherlands

**Keywords:** bilingualism, bilingual advantage, minority language, verbal working memory, exposure

## Abstract

Various studies have shown that bilingual children need a certain degree of proficiency in both languages before their bilingual experiences enhance their executive functioning (EF). In the current study, we investigated if degree of bilingualism in Frisian-Dutch children influenced EF and if this effect was sustained over a 3-year period. To this end, longitudinal data were analyzed from 120 Frisian-Dutch bilingual children who were 5- or 6-years-old at the first time of testing. EF was measured with two attention and two working memory tasks. Degree of bilingualism was defined as language balance based on receptive vocabulary and expressive morphology scores in both languages. In a context with a minority and a majority language, such as the Frisian-Dutch context, chances for becoming proficient in both languages are best for children who speak the minority language at home. Therefore, in a subsequent analysis, we examined whether minority language exposure predicted language balance and whether there was a relationship between minority language exposure and EF, mediated by language balance. The results showed that intensity of exposure to Frisian at home, mediated by language balance, had an impact on one of the attention tasks only. It predicted performance on this task at time 1, but not at time 2 and 3. This partially confirms previous evidence that the cognitive effects of bilingualism are moderated by degree of bilingualism and furthermore reveals that substantial minority language exposure at home indirectly affects bilingual children’s cognitive development, namely through mediation with degree of bilingualism. However, the findings also demonstrate that the effect of bilingualism on EF is limited and unstable.

## Introduction

The benefits of being proficient in two languages extend beyond the domain of language itself. Various studies have shown that bilingualism improves executive functioning (EF) (Adesope et al., 2010), a term which covers a broad range of cognitive functions that are used to control and regulate actions and thought (Miyake et al., 2000). Previous findings show that the cognitive effects of bilingualism are not found in all bilinguals, but require a sufficient degree of bilingualism (e.g., Bialystok and Barac, 2012).

In a bilingual context with two majority languages, more or less equal exposure to both languages at home provides the best basis for becoming a proficient bilingual. For example, in Quebec, children who had been exposed equally to French and English scored similarly to monolingual children on receptive vocabulary tests in each language (Thordardottir, 2011). However, in a bilingual context with a minority and a majority language the situation is different. A minority language is a language that is different from the language used by the majority of the inhabitants of a given country and that is spoken by a non-dominant group, who wish to maintain their own linguistic, and usually also cultural, identity (Hogan-Brun and Wolff, 2003). In such a context, a larger amount of home input in the minority language improves the chances for a high degree of bilingualism. For example, in Wales, all children become proficient speakers of English, regardless of their home language situation. Proficiency in Welsh, in contrast, depends on the amount of input in Welsh at home and at school (Gathercole and Thomas, 2009). In the United States, Spanish-English bilingual children’s development of Spanish receptive vocabulary is influenced by the amount of input at home, whereas this is not the case for the development of English receptive vocabulary (Hammer et al., 2009).

In the current study, we first investigated whether there is an effect of degree of bilingualism on EF in a group of Frisian-Dutch bilingual children, and whether this effect is maintained over time. Second, we examined whether there is an effect of Frisian exposure on EF that is mediated by degree of bilingualism. In what follows, we will first introduce the debate on bilingualism and EF. Subsequently, we will provide more information about the Frisian-Dutch bilingual context.

### Cognitive Effects of Bilingualism

Several studies have shown that bilingual children outperform monolingual children on EF (Adesope et al., 2010). Two EF components that have been found to be enhanced in bilinguals are attention (Martin-Rhee and Bialystok, 2008; Engel de Abreu et al., 2012) and working memory (Morales et al., 2013; Blom et al., 2014). Attention is the ability to focus on category-relevant aspects of the stimuli while ignoring category-irrelevant ones (Gazzaley and Nobre, 2012). Working memory refers to the capacity to store and manipulate information (Baddeley, 2007). The mechanism that is argued to lead to enhancement of EF in bilinguals is the monitoring of two co-activated languages in the brain. According to some researchers, the central process of this mechanism is inhibition of interference from the non-target language (Green, 1998), whereas others suggest that it is attention to the target language (Costa et al., 2006; Chung-Fat-Yim et al., 2016). In any case, it is argued that this linguistic practice of inhibition/attention generalizes to other, non-linguistic, domains, resulting in the bilingual EF advantage (Green, 1998; Bialystok et al., 2004; Costa et al., 2009; Chung-Fat-Yim et al., 2016). Previous studies have also found cognitive effects of bilingualism in majority-minority language settings with closely related languages, such as Italian and Sardinian (Lauchlan et al., 2013; Garraffa et al., 2015) and Cypriot Greek and Standard Modern Greek (Antoniou et al., 2016).

Although many studies have reported cognitive effects of bilingualism, these effects are not consistently replicated (e.g., Antón et al., 2014; Duñabeitia et al., 2014; Gathercole et al., 2014), thus calling into question the robustness of the bilingual advantage (Hilchey and Klein, 2011; Paap et al., 2015; Valian, 2015; Ross and Melinger, 2016). The inconsistencies in the literature have led some researchers to argue that the cognitive effects of bilingualism either do not exist or are restricted to very specific circumstances that pair the right set of bilingual experiences (Paap et al., 2015).

Most research on this topic has taken a cross-sectional approach, comparing monolinguals to bilinguals at one single point in time. However, group comparisons can never completely exclude the possibility of confounds (Woumans and Duyck, 2015). For example, as monolinguals and bilinguals often come from different cultural backgrounds, it can be difficult to disentangle effects of culture (Sabbagh et al., 2006; Oh and Lewis, 2008) from effects of bilingualism (but see Antón et al., 2014; Duñabeitia et al., 2014; Gathercole et al., 2014). As confounds can lead to misinterpretations, this is a potential reason for inconsistencies in the literature.

One way to overcome the problem of confounds is to avoid group comparisons and to treat bilingualism as a continuous, rather than as a binary variable. After all, bilingualism is not a matter of all or none, but comes in different degrees (Luk and Bialystok, 2013). Treating bilingualism as a gradient furthermore allows investigating if the effect of bilingualism on EF is moderated by degree of bilingualism. As the bilingual cognitive advantage is argued to arise from maintaining attention to the appropriate language system, the extent of this advantage should depend on how much effort is needed to monitor the two language systems. Since bilinguals with equal proficiency in both languages have to deal with a more active second language than bilinguals with unequal proficiency, it is thought that bilinguals with equal proficiency need more effort to maintain attention to the appropriate language system (Yow and Li, 2015).

### Defining Degree of Bilingualism

Various studies with children found support for the effect of degree of bilingualism on EF (Bialystok and Barac, 2012; Poarch and Van Hell, 2012; Videsott et al., 2012; Blom et al., 2014; Tse and Altarriba, 2014; Crivello et al., 2016; Prior et al., 2016; Thomas-Sunesson et al., 2016; Bosma et al., 2017). While some of these studies defined degree of bilingualism in terms of language balance (Prior et al., 2016; Thomas-Sunesson et al., 2016; Bosma et al., 2017), other studies defined it in terms of bilingual proficiency (Videsott et al., 2012; Blom et al., 2014; Tse and Altarriba, 2014; Crivello et al., 2016). Bilingual proficiency refers to the absolute and relative level of proficiency in both languages, while language balance only concerns the relative proficiency. These two constructs are related, because a high degree of bilingual proficiency implies a high degree of balance. However, they are not the same, because the reverse is not true. A high degree of balance does not necessarily imply a high degree of bilingual proficiency, since a child can be balanced with poor proficiency in both languages.

Following Yow and Li’s (2015) argument, balanced bilingual children with low proficiency in both languages are also thought to benefit from their bilingualism. This is one reason to define degree of bilingualism in terms of language balance rather than bilingual proficiency. Another reason is that previous research has shown that language proficiency in monolingual children also predicts EF (Hughes and Ensor, 2007; Fuhs and Day, 2011; Bohlmann et al., 2015; Kuhn et al., 2016), an observation that has so far not been taken into account in studies on the cognitive effects of bilingualism (but see Bohlmann et al., 2015). However, it implies that defining degree of bilingualism in terms of bilingual proficiency could create the risk that an observed effect of bilingual proficiency on EF is not an effect of bilingualism, but (partially) an effect of language proficiency that is independent of bilingualism. Therefore, it may be better to define degree of bilingualism in terms of language balance, because this measure does not include language proficiency.

In a recent study based on a subsample of the Frisian-Dutch bilingual children in the current study, we found that a group of 5- and 6-year-old balanced bilingual children outperformed a group of Dutch-dominant bilingual peers on a selective attention and a verbal working memory task, but not on an interference suppression and a visual working memory task (Bosma et al., 2017). In this previous study, children from the same classroom were assigned to either a balanced or a Dutch-dominant group. These two groups were matched on age, socioeconomic status (SES), non-verbal IQ scores and Dutch language abilities. By selecting matched groups we could exclude confounding variables, but also reduced the sample size and lost the precision of graduality. Therefore, in the present study, the full sample was included and degree of bilingualism was defined as a continuous variable. In doing so, we followed other studies in which children’s degree of bilingualism was defined in one of the following ways: as L2 proficiency (Tse and Altarriba, 2014), as the length of time in an immersion program (Bialystok and Barac, 2012), as a formula for language balance based on children’s receptive vocabulary scores in both languages (Thomas-Sunesson et al., 2016), as a formula for bilingual proficiency based on children’s receptive vocabulary scores in both languages (Blom et al., 2014), or as growth in the number of non-cognate translation equivalents between two measurements (Crivello et al., 2016). All these studies showed that degree of bilingualism predicts performance on EF tasks.

The present study extended previous research by investigating if the effect of degree of bilingualism was maintained over time. Since children’s linguistic and cognitive skills are still developing, it is possible, or even likely, that the cognitive effects of bilingualism are not stable. For example, Blom et al. (2014) found bilingual proficiency to predict verbal working memory at age 6, but not at age 5. As the children became more proficient in both languages between the ages of 5 and 6, this suggests that enhanced EF emerged as the children became more bilingually proficient. In contrast to Blom et al. (2014) we did not define degree of bilingualism in terms of bilingual proficiency, but in terms of language balance. As we have argued above, this is a slightly different measure. The children who participated in the present study were followed over a period of 3 years, starting with 5- and 6-year-olds. Previous findings of enhanced EF in bilinguals cover the whole age range of our study, from 5- and 6-year-olds (Blom et al., 2014; Gathercole et al., 2014; Tse and Altarriba, 2014; Prior et al., 2016; Bosma et al., 2017) to 7- and 8-year-olds (Bialystok and Barac, 2012; Engel de Abreu et al., 2012; Gathercole et al., 2014; Thomas-Sunesson et al., 2016), but the present study is, to our knowledge, the first that uses a 3-year longitudinal design to investigate the development of the effect of bilingualism on EF.

### Frisian-Dutch Bilingual Context

Frisian is a regional minority language that is spoken in the Dutch province of Fryslân, where it has official status next to the national majority language Dutch. Outside of the Netherlands, Frisian is known as West Frisian, to avoid confusion with the Frisian languages that are spoken in Germany. In this study, Frisian refers to West Frisian.

In 1998, the European Charter for Regional and Minority Languages (ECRML) went into force. With a recognition of the Frisian language under part III of this charter the Dutch government is obliged to take concrete actions to promote Frisian in domains like education, administration, and the media. For example, primary schools in Fryslân are required to teach Frisian as a subject for at least 1 h per week and in many schools Frisian is used as one of the languages of instruction. In 2005, the Dutch government recognized the Frisians as the only national minority group under the Framework Convention on the Protection of National Minorities (FCNM). Finally, in 2014, Frisian was recognized as official language of the province of Fryslân, next to Dutch, when the *Wet Gebruik Friese Taal* (‘Law on the use of the Frisian language’) went into force in the Netherlands.

The province of Fryslân has approximately 650.000 inhabitants (Centraal Bureau voor de Statistiek, 2017). Although Frisian is predominantly spoken in informal domains and more in rural than in urban areas (Breuker, 2001), it still has quite a strong position in the province as a whole. In a recent survey, a little more than half of the population reported to speak Frisian as a mother tongue (55.3%) and a little less than half of the population reported to speak Frisian with their partner (45.6%) and children (47.5%). Furthermore, the survey shows that Frisian is used more as an oral than as a written language: while the majority of the population reported to speak Frisian well (66.6%), only a small minority reported to write it well (14.5%) (Provinsje Fryslân, 2015).

Frisian and Dutch are both West Germanic languages. Historically, Frisian is most closely related to English, but over time English and Frisian have diverged, while Dutch and Frisian have converged (Gooskens and Heeringa, 2004). As a result, the Frisian and Dutch language that are spoken nowadays share a large part of their vocabularies and morphosyntactic structures. However, there are still quite a number of lexical and structural differences which clearly distinguish the two varieties.

Several studies have investigated how children’s proficiency in Frisian and Dutch develops before and during primary school and how this is related to home language exposure. Dijkstra (2013) showed that preschoolers with Frisian at home and preschoolers with Dutch at home (2.5- to 4-year-olds) performed similarly on a number of Dutch language measures, namely receptive vocabulary, mean length of utterance and number of different words. The only Dutch language task for which home language did matter was productive vocabulary. On the Frisian equivalents of all these tasks, the children with Frisian at home outperformed their peers with Dutch at home. Ytsma (1999) tested children’s Frisian and Dutch proficiency on a range of language tasks at the beginning and end of the first year of primary school (4- and 5-year-olds). The results showed that the children with Frisian at home progressed more in Dutch than the children with Dutch at home progressed in Frisian. By the end of the first year, the former group of children was more balanced in their two languages than the latter group of children. Van Ruijven (2006) showed that by the fourth year of primary school (7- and 8-year-olds), children with Frisian at home had caught up in Dutch language proficiency relative to their monolingual Dutch peers in the rest of the Netherlands. However, as Ytsma (1995) showed, children with Dutch at home did not catch up in Frisian relative to their peers with Frisian at home. Although most Dutch children did acquire some lexical knowledge of Frisian, they experienced great difficulty with the acquisition of the more structural aspects of the language, such as verb conjugation.

From the studies described above it is clear that in the Frisian-Dutch situation, children with Frisian at home have a good chance to become proficient bilinguals, whereas this is unlikely for children with Dutch at home. However, in these studies, language exposure was defined as a binary variable, either Frisian or Dutch, whereas in practice, most children are exposed to both languages at home, albeit in different relative amounts. Therefore, we investigated to what extent intensity of exposure to Frisian at home, defined as a gradient, predicts language balance. Subsequently, we investigated whether intensity of exposure to Frisian at home also predicts EF and if this effect is mediated by language balance. Exploring these relationships would provide more insight into the child-external factors that influence EF and the mechanism through which this can occur.

## Research Questions and Hypotheses

In the current study, we investigated the relationship between EF, exposure and degree of bilingualism in terms of language balance. The research questions are formulated in (1) and (2).

(1) Does degree of bilingualism predict Frisian-Dutch bilingual children’s performance on EF tasks that measure attention and working memory, and is this effect maintained over the course of 3 years?
(2) Does intensity of exposure to Frisian at home predict EF and is this relationship mediated by degree of bilingualism?

With respect to the first research question, we expected EF to be influenced by degree of bilingualism (Bialystok and Barac, 2012; Blom et al., 2014; Tse and Altarriba, 2014; Thomas-Sunesson et al., 2016). As cognitive effects of bilingualism have been found across the whole age range covered in our study (Bialystok and Barac, 2012; Engel de Abreu et al., 2012; Blom et al., 2014; Tse and Altarriba, 2014; Prior et al., 2016; Thomas-Sunesson et al., 2016; Bosma et al., 2017), we expected an effect on all three measurements. However, as children’s cognitive and linguistic skills were still developing, the effect may not be stable. Furthermore, as the cognitive effects of bilingualism are not consistently replicated (Antón et al., 2014; Duñabeitia et al., 2014; Gathercole et al., 2014), our study may also show mixed results.

With respect to the second research question, we hypothesized that intensity of exposure would predict EF performance and that this relationship would be mediated by degree of bilingualism. In line with previous evidence that only children with Frisian as their home language become proficient in both Frisian and Dutch (Ytsma, 1995, 1999; Van Ruijven, 2006; Dijkstra, 2013), we hypothesized that intensity of exposure to Frisian at home would predict degree of bilingualism to a large extent. As we expected degree of bilingualism to predict EF (research question 1), we hypothesized that intensity of exposure to Frisian at home would also predict EF.

## Materials and Methods

### Participants

Primary schools in the countryside of the Dutch province of Fryslân were contacted for the recruitment of participants. The 14 schools that were willing to participate distributed consent forms and information folders among the parents of the children. We only tested children whose parents had signed the consent form. These children were tested annually for three consecutive years. They were 5 or 6 years old at time 1, 6 or 7 years old at time 2, and 7 or 8 years old at time 3. In the first year of the study, a total of 122 children were assessed. After the first wave of data collection, two children dropped out, leaving 120 children for the present study (61 girls, 59 boys).

**Table 1** provides an overview of the participants’ age, non-verbal IQ scores, SES and intensity of exposure to Frisian at home. As age (Best et al., 2009), IQ (Arffa, 2007; but see Ardila et al., 2000) and SES (Calvo and Bialystok, 2014) are found to be correlated with EF, these measures were included as control variables in the current study. Non-verbal IQ was measured with the subsets Matrices and Recognition of the *Wechsler Non-verbal Scale of Ability* (Wechsler and Naglieri, 2006), which was assessed in the first year of the study. Through a questionnaire, based on the *Questionnaire for Parents of Bilingual Children* (Tuller, 2015), parents provided information regarding their own educational level, their children’s intensity of exposure to both languages at home and their children’s language use with friends. The mean educational level of the father and the mother was used as a proxy of SES. Education was measured on a 9-point scale, ranging from no education (1) to university degree (9). Intensity of exposure to each language was measured as the mean percentage of input that the child received from his father, mother, siblings and other adults. Other adults were only included in this score if they looked after the child at least once per week. For each of these people, we wanted to know how often (s)he spoke each language to the child: ‘never’ (0%), ‘seldom’ (25%), ‘sometimes’ (50%), ‘usually’ (75%) and ‘always’ (100%). Language use with friends was measured by asking how often the child spoke each language to other children (s)he regularly played with: ‘never’ (0%), ‘seldom’ (25%), ‘sometimes’ (50%), ‘usually’ (75%) and ‘always’ (100%). Intensity of exposure to Dutch was 100% minus intensity of exposure to Frisian. The same applies to Dutch language use with friends.

**Table 1:** Descriptive characteristics of the participants.

	Mean (*SD*)	Range	Maximum possible score
Age at time 1	70 (7)	59–83	
Age at time 2	82 (7)	71–95	
Age at time 3	94 (7)	83–107	
IQ	106 (15)	73–144	144
SES	6.9 (1.3)	3.5–9	9
% FR home	63 (29)	0–100	100
% FR friends	42 (24)	0–100	100

*Age, age in months; IQ, intelligence quotient; SES, socioeconomic status; % FR home, intensity of exposure to Frisian at home; % FR friends, intensity of Frisian language use with friends.*

### Measures

#### Degree of Bilingualism

We defined degree of bilingualism as relative proficiency in Frisian and Dutch. As language proficiency not only includes vocabulary, but also grammar (Treffers-Daller, 2015), we took into account both a receptive vocabulary and an expressive morphology task to define language proficiency in each language.

Dutch receptive vocabulary was measured with the Peabody Picture Vocabulary Test-III-NL (PPVT-III-NL; Schlichting, 2005), which is the Dutch version of the PPVT-III (Dunn and Dunn, 1997). Frisian receptive vocabulary was measured with an adaptation of the PPVT-III-NL, which was developed for the purpose of this project (Bosma et al., 2016). In this receptive vocabulary task, children were presented sheets with four pictures from which they had to choose the one that best represented an orally presented word. In total, the PPVT-III-NL contains 17 sets of 12 items, and the sets are ordered by difficulty. For the present study, we only used the first 12 sets, that is, the first 144 items, as these sets suffice to measure the vocabulary knowledge of the children in our age range. To make sure that all children completed all items, we did not use basal and ceiling criteria.

Dutch morphology was assessed with the subtest Word Formation of the *Taaltoets Alle Kinderen* (‘Language assessment all children,’ Verhoeven and Vermeer, 2002). This expressive task contained 12 items testing noun plural formation and 12 items testing past participle formation. In both Dutch and Frisian, regular nouns are pluralized by adding the suffix *-en* (Dutch/Frisian *boek-boeken* ‘book’-‘books’) or the suffix *-s* (Dutch/Frisian *tafel-tafels* ‘table-tables’). Regular participles in Dutch are formed with the circumfix *ge_t/d* (*dansen-gedanst* ‘dance-danced’, *rennen-gerend*, ‘run-run’), while regular participles in Frisian are formed with the suffix -t/-d (*bakke-bakt* ‘bake-baked’, *draaie-draaid* ‘turn-turned’) or with the suffix -e (*dûnsje-dûnse* ‘dance-danced’), depending on the infinitival form. In addition to these regular noun plurals and participles, the two languages have different types of irregular forms. Some forms are regular in Dutch, but irregular in Frisian, or vice versa.

To elicit noun plurals, children were presented with pictures of objects and prompt sentences of the following type: *Dat is een X, dat zijn twee…* “This is an X, these are two…”. To elicit past participles, children were presented with pictures and prompt sentences like the following: *Rosita is een bal aan het gooien. Gisteren heeft zij ook al een bal…* “Rosita is throwing a ball. Yesterday she has also … a ball.” Both the noun plural and the past participle part of the task contained items with different degrees of regularity. Frisian morphology was tested with a comparable morphology task that was developed for the purpose of this project (Blom and Bosma, 2016).

For both the vocabulary and the morphology tasks, percentage scores were calculated. To create a language proficiency score for each language, the vocabulary and morphology percentage scores were averaged. These Frisian and Dutch proficiency scores were used to calculate children’s degree of bilingualism in terms of language balance. This was done by dividing the lowest score (either Frisian or Dutch) by the highest and multiplying by 100, so that 100% indicated perfect language balance and lower scores indicated less balance.

#### Attention Measures

One of the attention tasks tested selective attention, which is the ability to filter information and focus on task-relevant cues, while the other tested interference suppression, which is the ability to suppress interference from distracting stimuli pulling for a competing response. Selective attention was measured with the Sky Search task from the *Test of Everyday Attention for Children* (Manly et al., 1998). Instruction was given in Dutch and the children were given a practice sheet before the test began. The task consisted of an A3 sheet with 128 pairs of spaceships, 20 of which were identical. The children had to draw a circle around the identical spaceship pairs as fast as they could, while ignoring the non-identical spaceship pairs. The task was timed with a stop watch. After they had completed this first sheet, the children got a second A3 sheet on which only the 20 target spaceships were displayed. In this motor-control version of the test they had to encircle all pairs of displayed spaceships as fast as they could. The attention score of the Sky Search was calculated by subtracting the mean time per target (one identical pair of spaceships) of the second sheet from the mean time per target of the first sheet. In this way, differences between children could not be the result of differences in circle drawing speed. Note that lower scores in this task indicated better performance. In the first year of the study, there were four children who encircled fewer than 15 spaceships on the motor-control sheet. In line with the manual of the Sky Search task, they were excluded from the analysis.

Interference suppression was measured with the Flanker task from Engel de Abreu et al. (2012), who adapted the task from Rueda et al. (2004). On a laptop, children were shown a horizontal row of five equally spaced yellow fish. They had to ignore the flanking fish and focus on the fish in the middle. By pressing a left or right response button, they had to indicate the direction of this central fish. Half of the flanking fish swam in the same direction as the target fish (congruent condition), while the other half swam in the other direction (incongruent condition). Each trial started with a fixation cross in the middle of the screen, which was shown for 1000 ms. Then the row of fish was presented for 5000 ms or until a response was given by pressing a left or a right button. Instruction was given in Dutch and the test started with eight practice trials before the real test began. The real test consisted of two blocks of 20 trials in which congruent and incongruent trials were randomly presented. Reaction times (RTs) and accuracy were recorded. The following responses were excluded from the analyses (9.92% of trials at time 1, 5.17% at time 2, 3.50% at time 3): incorrect responses (*n* = 425 at time 1, *n* = 178 at time 2, *n* = 102 at time 3), correct responses with RTs below 200 ms (*n* = 4 at time 1, *n* = 3 at time 2, *n* = 0 at time 3) and correct responses with RTs above three standard deviations of children’s individual congruent (*n* = 27 at time 1, *n* = 31 at time 2, *n* = 33 at time 3) and incongruent means (*n* = 16 at time 1, *n* = 36 at time 2, *n* = 33 at time 3). We calculated the difference between the RTs of the incongruent trials and the RTs of the congruent trials, which is also known as the Flanker effect (mean RT_INCONGRUENT_ – mean RT_CONGRUENT_). RTs for incongruent trials are usually slower than RTs for congruent trials, because of interference from the distracting flanking fish. The difference between the congruent and incongruent conditions is thought to measure interference inhibition: the smaller the Flanker effect, the better a child’s ability to suppress interference. At time 1, there was one child who only had one correct response in the incongruent condition. This child was excluded from the sample, as his mean RT for the incongruent condition could not be calculated reliably. At time 2 and 3, no children were excluded from the sample.

#### Working Memory Measures

Verbal working memory was measured with the Backward Digit Span task and visuospatial working memory with the Backward Dot Matrix task. These measures were based on the *Alloway Working Memory Assessment* (AWMA; Alloway, 2012) and translated to Dutch. In the Backward Digit Span, sequences of digits were auditorily presented and the children had to repeat them in reverse order. Since Dutch is the main language of education and all children had spent at least 1 year in education at the first time of testing, it was assumed that all children were able to count to ten in Dutch. In the Backward Dot Matrix, sequences of blue dots were presented in a 4 × 4 matrix on a computer screen. Each dot appeared on the screen for 2 s and when the dots had disappeared children were asked to point out the position of the dots in reverse order. For scoring, the AWMA procedure was applied. Per block, there was a maximum score of 6 points. When the child repeated the first four trials within one block correctly, he or she automatically continued with the next block and received a score of 6. After three incorrect trials within one block the task stopped. Trials were scored as incorrect if the sequence was incorrect, if children recalled one or more digits/dots incorrectly, or if they omitted one or more digits/dots. The scores could range from 0 to 36 for the Dot Matrix and from 0 to 42 for the Digit Span, so there were 6 and 7 blocks, respectively. In the first year of the study, the Backward Dot Matrix was aborted too early for one child. As this made the score unreliable, this data point was excluded from the analysis.

### Procedure

The tasks in this study were part of a larger test battery that included (language) tasks that were not reported on in the current study. They were administered in the following order, divided over two sessions of about 60 min each: Frisian receptive vocabulary, Frisian morphology, Digit Span, Sky Search and Flanker in the first session; Dutch receptive vocabulary, Dutch morphology and Dot Matrix in the second session. Children were tested in a quiet room at school, except for one child at time 1, four children at time 2 and five children at time 3, who were tested at home. The children were tested by the first author and two research assistants, who all had a native level command of both Dutch and Frisian.

## Results

### Descriptive Statistics

The mean scores and standard deviations of the language measures, degree of bilingualism and the cognitive measures are presented in **Table 2**. The vocabulary and morphology scores represent percentages correct, based on 144 and 24 items, respectively. Correlations between Frisian vocabulary and morphology scores ranged between *r*(120) = 0.442, *p* < 0.001, and *r*(120) = 0.514, *p* < 0.001. Correlations between Dutch vocabulary and morphology scores ranged between *r*(120) = 0.260, *p* = 0.004, and *r*(120) = 0.533, *p* < 0.001. Repeated measures ANOVAs showed that over time, children improved on all language measures, *p* < 0.001. LSD *post hoc* tests showed that the differences between Time 1 and Time 2 and between Time 2 and Time 3 were significant at the *p* < 0.001 level for all language measures. Degree of bilingualism in terms of language balance is based on Dutch and Frisian receptive vocabulary and morphology scores with a score of 100% representing perfect language balance. A repeated measures ANOVA showed that on average, degree of bilingualism did not change over time, *p* = 0.267, $\eta_{p}^{2}$ = 0.011. However, as they grew older, more children became dominant in Dutch, 55.8% at time 1, 64.2% at time 2, 75.8% at time 3.

**Table 2:** Descriptive statistics of the language and cognitive measures.

	Time 1 *M* (*SD*)	Time 2 *M* (*SD*)	Time 3 *M* (*SD*)	*p*	η^2^
**Language measures**					
Dutch receptive vocabulary	64.38 (5.34)	69.87 (4.51)	73.49 (4.94)	<0.001	0.689
Dutch morphology	63.54 (13.28)	75.07 (12.52)	85.38 (10.23)	<0.001	0.699
Frisian receptive vocabulary	62.91 (6.33)	68.03 (5.62)	71.68 (5.17)	<0.001	0.636
Frisian morphology	54.03 (22.52)	60.69 (23.35)	65.63 (23.15)	<0.001	0.324
Degree of bilingualism	82.21 (14.72)	82.98 (15.27)	83.39 (14.75)	0.267	0.011
**Cognitive measures**					
Sky Search	10.39 (7.50)	5.54 (2.63)	4.10 (1.61)	<0.001	0.414
Flanker effect	225 (299)	123 (174)	93 (172)	<0.001	0.097
Backward digit span	12.81 (2.87)	14.90 (2.88)	16.47 (3.57)	<0.001	0.391
Backward dot matrix	13.27 (4.76)	18.16 (4.55)	20.69 (4.68)	<0.001	0.569

For the Sky Search and the Flanker effect, lower scores indicate better performance, whereas for the Backward Digit Span and the Backward Dot Matrix, higher scores indicate better performance. Repeated measures ANOVAs showed that over time, children significantly improved on all four cognitive measures, *p* < 0.001. LSD *post hoc* tests showed that for the Sky Search, Digit Span and Dot Matrix, the differences between Time 1 and Time 2 and between Time 2 and Time 3 were significant at the *p* < 0.001 level. For the Flanker, the difference between Time 1 and Time 2 was also significant, *p* = 0.001, but the difference between Time 2 and Time 3 was not, *p* = 0.087. Correlations between age in months, IQ, SES, intensity of exposure, degree of bilingualism and the cognitive measures at time 1, 2, and 3 are reported in **Tables 3–5**, respectively.

**Table 3:** Correlations between age, IQ, SES, intensity of exposure to Frisian at home, degree of bilingualism and the cognitive measures at time 1.

	IQ	SES	% FR	DegBil	Sky Search	Flanker	BW digit	BW dot
Age	–0.020	–0.118	0.098	0.094	–0.300***	0.025	0.264**	0.271**
IQ	–	0.039	–0.007	0.058	–0.236*	–0.155	0.363***	0.285**
SES		–	–0.244**	–0.095	0.057	–0.045	–0.003	0.101
% FR			–	0.682***	–0.220*	0.050	0.055	–0.052
DegBil				–	–0.245**	–0.033	0.143	0.061
Sky Search					–	0.104	–0.300***	–0.263**
Flanker						–	–0.244**	–0.191*
BW digit							–	0.438***

*% FR, intensity of exposure to Frisian at home; DegBil, degree of bilingualism. ^∗^*p* ≤ 0.05; ^∗∗^*p* ≤ 0.01; ^∗∗∗^*p* ≤ 0.001.*

**Table 4:** Correlations between age, IQ, SES, intensity of exposure to Frisian at home, degree of bilingualism and the cognitive measures at time 2.

	IQ	SES	% FR	DegBil	Sky Search	Flanker	BW digit	BW dot
Age	–0.026	–0.115	0.100	0.036	–0.352***	–0.164	0.126	0.268**
IQ	–	0.039	–0.007	0.048	–0.159	–0.015	0.265**	0.322***
SES		–	–0.244**	–0.154	–0.036	0.152	0.141	0.033
% FR			–	0.784***	–0.185*	–0.061	0.016	0.059
DegBil				–	–0.216*	–0.011	0.136	0.125
Sky Search					–	0.110	–0.064	–0.379***
Flanker						–	–0.013	–0.169
BW digit							–	0.448***

*% FR, intensity of exposure to Frisian at home; DegBil, degree of bilingualism. ^∗^*p* ≤ 0.05; ^∗∗^*p* ≤ 0.01; ^∗∗∗^*p* ≤ 0.001.*

**Table 5:** Correlations between age, IQ, SES, intensity of exposure to Frisian at home, degree of bilingualism and the cognitive measures at time 3.

	IQ	SES	% FR	DegBil	Sky Search	Flanker	BW digit	BW dot
Age	–0.028	–0.123	0.099	0.014	–0.260**	–0.081	0.102	0.296***
IQ	–	0.039	–0.007	0.070	–0.114	–0.238**	0.332***	0.283**
SES		–	–0.244**	–0.166	–0.004	–0.131	0.151	0.089
% FR			–	0.812***	–0.045	0.046	–0.001	0.084
DegBil				–	–0.057	0.141	–0.003	0.074
Sky Search					–	0.365***	–0.145	–0.241**
Flanker						–	–0.059	–0.201*
BW digit							–	0.431***

*% FR, intensity of exposure to Frisian at home; DegBil, degree of bilingualism; ^∗^*p* ≤ 0.05; ^∗∗^*p* ≤ 0.01; ^∗∗∗^*p* ≤ 0.001.*

### The Effect of Degree of Bilingualism on EF

The first research question of this study was whether degree of bilingualism predicts EF and whether this effect is stable over the course of 3 years. The correlation matrices in **Tables 3–5** show that degree of bilingualism correlated with one of the four cognitive tasks, namely the Sky Search task. Therefore, follow-up regression analyses were performed for this task only. The correlation matrices also show that the Sky Search task significantly correlated with age and IQ, but not with SES. Therefore, only age and IQ were included as control variables in the regression analyses. As the distribution of the Sky Search task deviated strongly from normality (time 1: skew = 2.33, kurtosis = 7.07; time 2: skew = 2.49, kurtosis = 9.83; time 3: skew = 2.68, kurtosis = 10.43), we applied a log-transformation to improve the distribution (time 1: skew = 0.37, kurtosis = 0.00; time 2: skew = 0.70, kurtosis = 0.89; time 3: skew = 0.93, kurtosis = 1.99). Three sequential hierarchical multiple regression analyses were conducted with the Sky Search task at time 1, time 2 and time 3 as dependent variables. In the first step of the model, age and IQ were included as control variables. In the second step of the model, degree of bilingualism at the time of testing was added as a predictor. The results are shown in **Table 6**. Degree of bilingualism predicted performance on the Sky Search task at time 1, β = -0.191, *p* = 0.026, but not at time 2, β = -0.163, *p* = 0.058, and time 3, β = -0.027, *p* = 0.764.

**Table 6:** Sky Search at time 1, 2 and 3, regressed on degree of bilingualism at the time of testing, controlling for age, IQ and SES.

	Sky Search Time 1 (*n* = 116)		Sky Search Time 2 (*n* = 120)		Sky Search Time 3 (*n* = 120)	
	Stage 1 (β)	Stage 2 (β)	Stage 1 (β)	Stage 2 (β)	Stage 1 (β)	Stage 2 (β)
Age Tx	–0.371***	–0.354***	–0.344***	–0.338***	–0.267**	–0.266**
IQ	–0.201*	–0.189*	–0.119	–0.110	–0.118	–0.116
Bilingualism Tx		–0.191*		–0.163		–0.027
*R*^2^	0.176	0.212	0.130	0.157	0.083	0.084
Δ*R*^2^		0.036		0.027		0.001
*F*	12.091***	10.058***	8.746***	7.180***	5.325**	3.552*

*Tx = time 1, 2 and 3, respectively; ^∗^*p* ≤ 0.05; ^∗∗^*p* ≤ 0.01; ^∗∗∗^*p* ≤ 0.001.*

### The Effect of Minority Language Exposure on EF

The second research question of this study was whether there is a relationship between intensity of exposure to Frisian at home and EF, mediated by degree of bilingualism.

In order to answer this question we first investigated to what extent intensity of exposure to Frisian at home predicted children’s degree of bilingualism. Second, we investigated whether intensity of exposure to Frisian at home predicted EF.

The correlation matrices in **Tables 3–5** show that degree of bilingualism and intensity of exposure to Frisian were highly correlated, but that degree of bilingualism did not significantly correlate with age, IQ and SES. Therefore, no control variables were included in the follow-up regression analyses. Three sequential hierarchical multiple regression analyses were conducted with degree of bilingualism at time 1, time 2 and time 3 as dependent variables and intensity of exposure to Frisian at home as predictor. The results (**Table 7**) showed that intensity of exposure to Frisian at home predicted degree of bilingualism to a large extent at time 1, β = 0.682, *p* < 0.001, time 2, β = 0.784, *p* < 0.001, and time 3, β = 0.812, *p* < 0.001.

**Table 7:** Degree of bilingualism at time 1, 2 and 3, regressed on intensity of exposure to Frisian at home.

	Bilingualism Time 1 (*n* = 120) (β)	Bilingualism Time 2 (*n* = 120) (β)	Bilingualism Time 3 (*n* = 120) (β)
Exposure FR	0.682^∗∗∗^	0.784^∗∗∗^	0.812^∗∗∗^
*R*^2^	0.465	0.614	0.659
*F*	102.604^∗∗∗^	187.722^∗∗∗^	227.846^∗∗∗^

*^∗^*p* ≤ 0.05; ^∗∗^*p* ≤ 0.01; ^∗∗∗^*p* ≤ 0.001.*

The correlation matrices in **Tables 3–5** show that intensity of exposure to Frisian at home correlated with the Sky Search task at time 1, *r*(116) = -0.220, *p* = 0.018, and time 2, *r*(120) = -0.185, *p* = 0.043. In order to further investigate this relationship we conducted three hierarchical multiple regression analyses with the Sky Search task at time 1, time 2 and time 3 as dependent variables. Again, we used the log-transformations of the Sky Search task. In the first step of the model, age and IQ were included as control variables. In the second step of the model, intensity of exposure to Frisian at home was added as a predictor. The results are shown in **Table 8**. Intensity of exposure to Frisian at home predicted performance on the Sky Search task in more or less the same way as degree of bilingualism did (research question 1). There was an effect at time 1, β = -0.171, *p* = 0.046, but not at time 2, β = -0.129, *p* = 0.137, and time 3, β = -0.015, *p* = 0.867.

**Table 8:** Sky Search at time 1, 2 and 3, regressed on intensity of exposure to Frisian at home, controlling for age, IQ and SES.

	Sky Search Time 1 (*n* = 116)		Sky Search Time 2 (*n* = 120)		Sky Search Time 3 (*n* = 120)	
	Stage 1 (β)	Stage 2 (β)	Stage 1 (β)	Stage 2 (β)	Stage 1 (β)	Stage 2 (β)
Age Tx	–0.371***	–0.352***	–0.344***	–0.331***	–0.267**	–0.265**
IQ	–0.201*	–0.202*	–0.119	–0.119	–0.118	–0.118
Exposure FR		–0.171*		–0.129		–0.015
*R*^2^	0.176	0.205	0.130	0.147	0.083	0.084
Δ*R*^2^		0.029		0.017		0.001
*F*	12.091***	9.631***	8.746***	6.642***	5.325**	3.530*

*Tx = time 1, 2, and 3, respectively; ^∗^*p* ≤ 0.05; ^∗∗^*p* ≤ 0.01; ^∗∗∗^*p* ≤ 0.001.*

Partial correlations controlling for degree of bilingualism showed no significant relationship between intensity of exposure to Frisian at home and the Sky Search task at time 1, *r*(116) = -0.073, *p* = 0.440, suggesting that the relationship between Frisian exposure and the Sky Search task at time 1 was indeed mediated by degree of bilingualism.

## Discussion

The first aim of the study was to examine whether degree of bilingualism has an effect on Frisian-Dutch bilingual children’s EF and whether this effect is maintained as the children grow older. Whereas most previous studies on the cognitive effects of bilingualism compared monolinguals to bilinguals (e.g., Engel de Abreu et al., 2012), the current study adds to the few studies in which children’s bilingualism is defined as a gradient (Bialystok and Barac, 2012; Blom et al., 2014; Tse and Altarriba, 2014; Crivello et al., 2016; Thomas-Sunesson et al., 2016), doing justice to the graduality of bilingualism (Luk and Bialystok, 2013). In the present study, we defined degree of bilingualism in terms of language balance. Our results partly confirmed previous research showing that bilingualism enhances EF (e.g., Adesope et al., 2010) and that the effects are moderated by language balance (Prior et al., 2016; Thomas-Sunesson et al., 2016; Bosma et al., 2017). However, the effect was limited to selective attention and disappeared over time, thus supporting previous skepticism about the robustness of the bilingual advantage (Hilchey and Klein, 2011; Paap et al., 2015; Valian, 2015; Ross and Melinger, 2016). There was a significant effect of degree of bilingualism on the Sky Search task at time 1 (age 5/6), a close to significant effect at time 2 (age 6/7) and no effect at time 3 (age 7/8). There was no effect on interference suppression, as measured with the Flanker task, and working memory, as measured with the Backward Digit Span and the Backward Dot Matrix.

The absence of an effect on working memory is in contrast with Bosma et al. (2017), who used a subsample of the children in the current study and found that balanced Frisian-Dutch bilingual children outperformed Dutch-dominant bilingual children on verbal working memory and selective attention. The absence of an effect on verbal working memory in the current study suggests that the effect of bilingualism on verbal working memory is less robust than the effect of bilingualism on selective attention. The finding that degree of bilingualism only had an effect on selective attention strengthens the view that selective attention, rather than interference suppression, is the core of the bilingual EF advantage (Chung-Fat-Yim et al., 2016). Chung-Fat-Yim et al. (2016) argue that the ability to selectively attend to visual stimuli and to disengage from the focus of attention when criteria are not met is similar to the kind of challenge that bilinguals face every day, namely to selectively attend to the linguistic structures of the target language and to disengage attention from structures that do not belong to the target language.

The second aim of the study was to investigate whether exposure to the minority language at home has an effect on EF and whether this effect is mediated by degree of bilingualism. Finding this relationship would provide more insight into the child-external factors that influence EF and the mechanism through which this can occur. Although many studies have investigated the circumstances that support bilingual language acquisition (e.g., Gathercole and Thomas, 2009; Hammer et al., 2009; Dijkstra, 2013), it has only rarely been investigated whether these circumstances indirectly lead to cognitive enhancement (but see Bialystok and Barac, 2012). The results of our study showed that intensity of exposure to Frisian at home predicted degree of bilingualism to a large extent, a finding that is in line with previous evidence that in the province of Fryslân, only children with Frisian as their home language become proficient bilinguals (Ytsma, 1995, 1999; Van Ruijven, 2006; Dijkstra, 2013). Furthermore, intensity of exposure to Frisian at home predicted EF in the same way as degree of bilingualism did, that is, there was an effect on the Sky Search task at time 1, but not at time 2 and 3.

The current study is the first study that examined the effect of language balance on EF in a longitudinal way. The finding that the effect on selective attention fluctuates over time is important, because it may explain some inconsistencies in the literature. Namely, if the current study were cut into three separate cross-sectional studies, these three studies would have contradicted each other, as only one out of three would have found an effect. By following the same group of children for a longer period of time, we were able to show the instability of the cognitive effect of bilingualism. One possibility for the vanishing of the effect is that Dutch is the dominant language in school, which would lead to a reduction in the use of the minority language as the children grow older. This is also supported by the finding that over time, children became more dominant in Dutch. However, as children’s overall language balance did not change over time, other explanations may be more likely.

As Valian (2015) pointed out, the cognitive effects of bilingualism may not always be visible, because they are very small and probably compete with many other activities that also enhance EF. Following this line of reasoning, one alternative explanation for the vanishing of the effect is that over time, the effect of bilingualism on visual selective attention got overruled by the effect of literacy. Several studies have shown that literacy enhances visual discrimination abilities (e.g., Ventura et al., 2013; Pegado et al., 2014). Pegado et al. (2014), for example, showed that learning to read has an impact on several stages of visual processing, including repetition suppression. This is the reduction in neural activity in response to a repeated stimulus. Since repetition suppression reflects the brain’s capacity to discriminate two items, this suggests that literacy facilitates the identification of identical visual stimuli, a skill that is useful for the Sky Search task. In the first year of our study, most children were in grade 2 and had not started formal literacy education yet. However, by the third year of our study, all children had received between 0.5 and 2.5 years of literacy instruction. It could be that the age at which formal literacy instruction begins influences when the cognitive effects of bilingualism are visible. Therefore, we suggest that future studies investigate the cognitive effects of bilingualism in combination with the cognitive effects of literacy.

Another potential reason why the effect disappears over time is given by Gathercole et al. (2014). They argue that links within a language are usually stronger than links across languages. However, in fluent bilinguals, the between-language links are quite strong and as their linguistic knowledge in both languages is automatized, they may require little cognitive control to monitor their two co-activated languages. On average, the language balance of the children in our study did not improve over time, but their proficiency in Dutch and Frisian did. Following Gathercole et al.’s (2014) line of reasoning, the children in our study with a high degree of language balance may have strengthened the links between their two languages as they grew older, which might have resulted in the leveling off of the cognitive effect. While this explanation seems to be at odds with the suggestion that the effect of bilingualism develops as a result of growing bilingual proficiency (Blom et al., 2014), it is not impossible that once a higher degree of proficiency in both languages has been attained, bilingual monitoring becomes more automatic and bilingual experience does not further enhance EF. What this suggests is a limited window of development in which bilingualism enhances cognitive functioning.

Taken together, the current study only provides minimal support for the claim that minority language exposure, mediated by language balance, influences the cognitive effects of bilingualism. The effect was only visible on one out of four EF tasks and disappeared as the children grew older, thus supporting previous skepticism about the robustness of the bilingual advantage (Hilchey and Klein, 2011; Paap et al., 2015; Valian, 2015; Ross and Melinger, 2016). Although the reasons for this fluctuation over time remain as yet unclear, the instability of the effect may explain why some cross-sectional studies show cognitive enhancement in bilinguals, whereas other studies do not.

## Ethics Statement

All the parents of the participating children gave their written informed consent, as was stated in the Section “Materials and Methods” of our paper. Unfortunately, the study was not officially evaluated by an ethics committee before the start of the study due to a miscommunication. In hindsight, the ethics committee of the University of Amsterdam evaluated the information folder and the informed consent form that we used and came to the conclusion that the research had been conducted with the wellbeing of the participants in mind.

## Author Contributions

All authors listed have made a substantial, direct and intellectual contribution to the work, and approved it for publication.

## Conflict of Interest Statement

The authors declare that the research was conducted in the absence of any commercial or financial relationships that could be construed as a potential conflict of interest.
